# Incidents and adverse events notification system in a post-cardiac surgery unit. initial experience

**DOI:** 10.1186/2197-425X-3-S1-A74

**Published:** 2015-10-01

**Authors:** P Ruiz de Gopegui Miguelena, CI Bernal-Matilla, P Sánchez-Chueca, R Ramos-Abril, AL Ruiz-Aguilar, F Barra-Quílez, J Navarro, D Mellado, B Vicente, A Tejada-Artigas

**Affiliations:** Intensive Care Service, Hospital Universitario Miguel Servet, Zaragoza, Spain

## Introduction

Search for patient safety is mandatory for improvements in ethical and quality in intensive care units. Participation of all healthcare professional categories is required to achieve the highest degree in patient safety so that a Patient Safety Group (PSG) was founded to conduct policies in the Intensive Medicine Service (IMS).

## Objectives

To implement an incidents and adverse events notification system (IAENS) in a Post-Cardiac Surgery Unit (PCSU) part of an Intensive Medicine Service (IMS).

## Methods

PSG developed an IAENS in February 2014. After its approval by Hospital Ethical and Clinical Security Committees it was implemented in the PCSU on April as first essay before its generalization to the whole IMS. IAENS consists in a voluntary and anonymous report of adverse events (AE) in paper form by IMS staff and related healthcare professionals. A prospective study of reported AE was performed for the period March 2014 to January 2015. Collected data about AE were date, place and nurse shift at which happened, free text report, number of patient admitted and nurse: patient ratio at that time, associated factors and classification in 12 groups (medication, airway, procedure, nosocomial infection, etc.), severity and preventability. We also collected data about patient (sex, age, length of stay, etc.) and professional category of the person who reported.

## Results

During the study period 513 patients were admitted; none of them suffered an AE as a cause for PCSU admission. 117 AE were reported, 30% in March, affecting to 75 patients mean age 67.96 years, 77% males, and most of them were discharged to ward (97.5%). Mean admission days until the happening of an AE were 7.68 ± 30.1 days. PCSU overall mean stay were 16.45 ± 25.65 days. Most of the EA were reported by nurse staff (61.5%), during the morning shift (43.6%) with total occupancy of PCSU (73'5%). The most important group of AE (30%) was “unanticipated disconnection or withdrawal of catheter, probe, tube, drainage or sensor” and near the half (49'6%) were considered undoubtedly preventable. The third part (32.5%) of the affected patients required any kind of monitoring to assure that the AE did not damaged them. Only 5.1% of the AE compromised the life of the patients.

## Conclusions

As the IAENS consists in voluntary report we consider that the number of AE reported is a small part of the total AE suffered by patients. There were less than one reported AE per day and very few patients were affected. Although the damage caused were not serious we must point out that the most of EA are considered preventable so the PSG should develop policies to avoid related risk factors.

## References

[1] Merino P (2012). Adverse events in Spanish intensive care units: the SYREC study. Int Qual Health Care.

